# Liprin-α family proteins and their cellular functions

**DOI:** 10.1016/j.jbc.2026.113183

**Published:** 2026-05-25

**Authors:** Abigail Mayer, Yang Zhang, Houhui Xia

**Affiliations:** 1 Neuroscience Graduate Program, Department of Neuroscience, University of Rochester Medical Center, Rochester, New York, USA; 2 Department of Pharmacology and Physiology, University of Rochester Medical Center, Rochester, New York, USA

**Keywords:** active zone, cell signaling, focal adhesion, liprin-α, liquid-liquid phase separation, Phosphorylation, protein-protein interaction, scaffolding protein, Synapse, synaptic transmission

## Abstract

Scaffold proteins are critical for the spatial organization and assembly of subcellular compartments through interactions with specific binding partners. The liprin-α family of scaffold proteins are implicated in diverse cellular functions across both neuronal and non-neuronal cell types. All four liprin-α isoforms contribute to presynaptic active zone formation, promoting synaptic vesicle accumulation and neurotransmitter release. Additionally, there is evidence that liprin-α plays a role in postsynaptic cellular processes such as dendritic growth, spinogenesis, and AMPA receptor trafficking. Due to ubiquitous expression, liprin-α1 regulates cell adhesion and motility. This review summarizes liprin-α functions, emphasizing its interactors and associated signaling pathways. Liprin-α proteins participate in multivalent interactions and undergo liquid-liquid phase separation, a mechanism postulated to promote the formation of membrane-less compartments. This review further examines regulatory mechanisms that govern liprin-α function and phase separation.

Protein scaffolds are fundamental to the spatial organization of protein-protein interactions that facilitate precise and efficient cellular signaling across diverse cell types. The liprin-α family of scaffolding proteins play a critical role in regulating synapse assembly and maturation in neurons and are also essential for the formation of focal adhesions and the regulation of cell motility in non-neuronal cells (1, 2). In mammals, four highly conserved liprin-α isoforms (liprin-α1 to liprin-α4) have been identified, each exhibiting distinct expression patterns (3, 4). Liprin-α1 is ubiquitously expressed, whereas liprin-α2, liprin-α3, and liprin-α4 are predominantly expressed in the brain, with liprin-α4 also detected in cardiac and skeletal muscle tissues. Invertebrates express a single ortholog of mammalian liprin-α, termed SYD-2 in *Caenorhabditis elegans* and Dliprin-α in *Drosophila* (5, 6).

Liprin-α isoforms share a conserved domain architecture, including an N-terminal coiled-coil domain and three tandem sterile alpha motifs (SAMs) at the C-terminus, also referred to as the liprin homology domain. Liprin-α was initially identified through its interaction with leukocyte common antigen-related receptor protein tyrosine phosphatases (LAR-RPTPs) (3, 7), with subsequent studies elucidating numerous additional binding partners (2). The N-terminal coiled-coil domain mediates both oligomerization and interactions with ELKs (ERC1), RIM, and GIT1 (8, 9, 10, 11), while the SAM domains facilitate binding to LAR-RPTPs, CASK, and other scaffolding proteins including liprin-β isoforms (3, 12, 13, 14, 15) (Fig. 1). Due to its ubiquitous expression, liprin-α1 has been extensively investigated across various biological contexts and studies have identified several liprin-α1 interactors, including activated integrin β1 (16), KIF7 (17), CEP170B (18), KIF1A (19), GRIP (20), and regulatory B subunits of protein phosphatase 2A (PP2A) (17, 21, 22, 23) (Fig. 2). These interactions underpin the pivotal roles of liprin-α proteins at the neuronal active zone and postsynaptic density, whereas liprin-α1 additionally contributes to focal adhesion dynamics (24), cell motility (25, 26, 27), microtubule regulation (18), and sonic hedgehog signaling (17) in non-neuronal cells.

**Figure 1:**
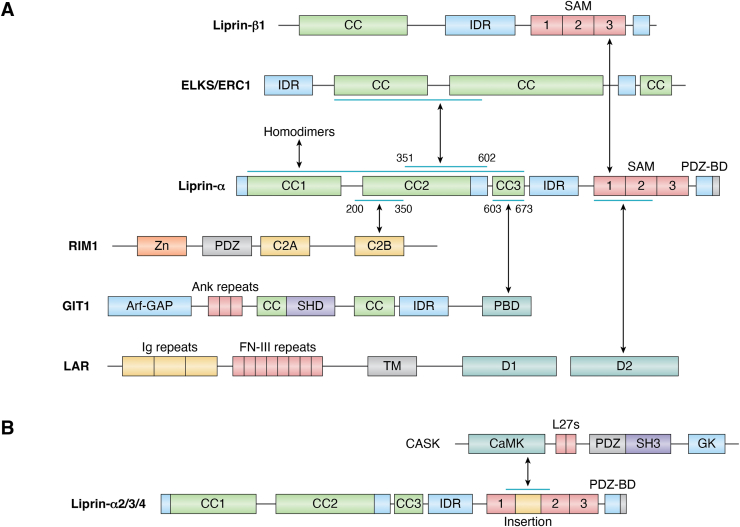
**Liprin-α interactors.***A*, schematic of liprin-α interaction with liprin-β1, ELKS/ERC1, RIM1, GIT1, LAR, and other liprin-α proteins to form homodimers. *B*, schematic of liprin-α2/3/4 interaction with CASK. *A* and *B*, *lines* and *arrows* indicate the domains responsible for interactions. If known, liprin-α residue numbers involved in interaction are listed. Ank Repeats, Ankyrin tandem repeats; Arf-GAP, Arf GTPase-activating protein domain; C2A, C2B, C2 domain; CaMK, calmodulin kinase domain; CC, Coiled coil domain; D1, D2, protein tyrosine phosphatase homology domain; FN-III repeats, fibronectin type III domains; GK, guanylate kinase domain; IDR, intrinsically disordered region; Ig repeats, immunoglobulin repeat domains; L27, L27 domain; PBD, paxillin-binding domain; PDZ, PDZ domain; PDZ-BD, PDZ-binding domain; SAM, sterile alpha motif; SH3, Src homology 3 domain; SHD, Spa2-homology domain; TM, transmembrane domain; Zn, Zinc finger structure. Figure created using BioRender.

**Figure 2:**
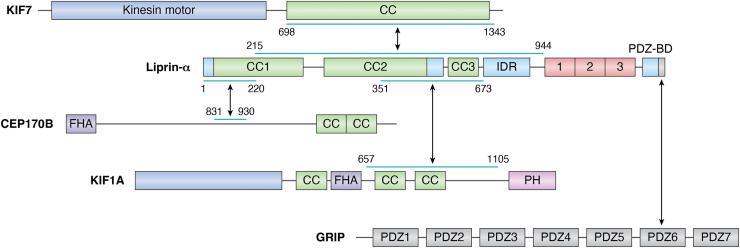
**Liprin-α1 interactors.** Liprin-α1 binds directly to kinesin motor proteins KIF7 and KIF1A as well as CEP170B and GRIP. *Lines* and *arrows* indicate the domains responsible for interactions. If known, liprin-α residue numbers involved in interaction are listed. CC, Coiled coil domain; IDR, intrinsically disordered region; PDZ, PDZ domain; PDZ-BD, PDZ-binding domain; SAM, sterile alpha motif. PDZ type II ligand sequence on Liprin-α1 is VRTYSC. FHA, Forkhead-Associated domain; PH, Pleckstrin Homology domain. Whether liprin-α1 binds to CASK *via* PDZ ligand-PDZ domain interaction is not known. Figure created using BioRender.

## Liprin-α promotes active zone component accumulation and neurotransmitter release

Studies utilizing invertebrate models suggested liprin-α proteins play significant roles in presynaptic assembly and morphology (5, 6, 8, 28). For example, *Drosophila* express a single homolog for liprin-α and LAR which are both essential for proper synapse morphology (5). Similarly, in *C. elegans*, the liprin-α ortholog SYD-2 is required for normal synapse structure and presynaptic protein localization (28). Electron microscopy revealed that SYD-2 regulates the size of the electron-dense projection, a key feature of the active zone, with loss of SYD-2 leading to smaller projections and gain-of-function causing enlargement. These findings establish liprin-α proteins as critical regulators of active zone formation and morphogenesis. However, given that mammalian neurons express four liprin-α isoforms, further investigation was needed to elucidate their specific roles in active zone assembly and function.

**Figure 3:**
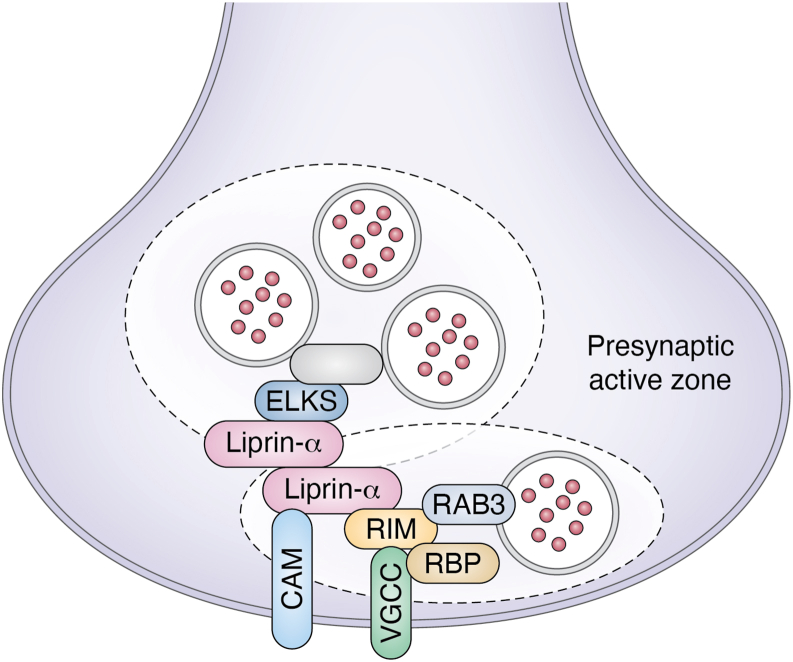
**Liprin-α presynaptic function.** Simplified schematic of liprin-α function at the presynaptic active zone. Liprin-α binds to cell adhesion molecules (CAMs) to localize to the active zone. Liprin-α then binds to RIM which promotes the localization of synaptic vesicles near voltage-gated calcium channels (VGCC). Liprin-α also binds to ELKS to accumulate synaptic vesicles at the active zone. *Dotted circles* represent proteins that have been shown to undergo phase separation together. Figure created using BioRender.

In mammalian systems, liprin-α isoforms have been implicated in presynaptic active zone assembly (29) (Fig. 3). Superresolution microscopy revealed that liprin-α3 exhibits strong co-localization with active zone markers, whereas liprin-α2 displays a more diffuse nerve terminal distribution (30). Single liprin-α3 knockout and liprin-α2/3 double knockout models exhibit impaired synaptic vesicle docking, exocytosis, and decreased synaptic abundance of liprin-α interactors such as RIM, CASK, and Munc-13 (30, 31, 32). Isoform compensation, notably increased liprin-α2 active zone localization following liprin-α3 loss, limits individual knockout interpretations (30). A more recent quadruple liprin-α knockout (qKO) model using human iPSC-derived glutamatergic neurons demonstrated profound mislocalization of presynaptic components and diminished synaptic transmission, confirming liprin-α proteins as master regulators of active zone assembly (29). Rescue experiments indicated functional redundancy among liprin-α isoforms. Collectively, these findings, corroborated by invertebrate data, establish liprin-α as a critical scaffold for active zone protein and synaptic vesicle recruitment as well as active zone morphogenesis.

The domain architecture of liprin-α and its protein interactions are well-defined, enabling targeted manipulation to elucidate its role in active zone assembly. Domain-specific mutants have been employed to assess the necessity of liprin-α interactions for its function (29). Liprin-α binding to presynaptic cell adhesion molecules, including LAR-RPTP and CASK, is essential for synaptic localization and proper active zone protein distribution. Mutants deficient in binding these interactors fail to localize to synapses, resulting in mislocalization of downstream active zone components. Furthermore, liprin-α interaction with ELKS is critical for ELKS accumulation at the active zone and subsequent recruitment of additional active zone proteins and synaptic vesicles (29, 33). Together, these findings support a model, wherein liprin-α binding to cell adhesion molecules constitutes an early step in active zone formation, facilitating the subsequent assembly of active zone proteins and synaptic vesicle clustering. Liprin-α also plays roles beyond active zone formation and contributes to calcium-triggered vesicle/neurotransmitter release *via* preferential enrichment of calcium channels in liprin-α2-RIM condensates (34), a distinct, non-stoichiometric membrane-less compartment within a cell (Fig. 3). Overall, the liprin-α family of proteins are crucial for active zone formation and function.

## Liprin-α promotes dendritic growth, spinogenesis, AMPAR trafficking, and synaptic plasticity

While liprin-α function was notably studied in the presynaptic terminal, it was also reported to localize to dendrites where liprin-α plays important roles in dendrite growth as well as postsynaptic density organization and function. Overall, there is evidence for liprin-α proteins contributing to neurite extension/dendrite growth, synapse formation, AMPAR trafficking, and synaptic plasticity (9, 13, 20, 35, 36, 37).

### Dendrite growth

Liprin-α1 plays critical roles in dendrite growth in glutamatergic (13, 36) and GABAergic neurons (35). Knocking down liprin-α1 reduced dendritic branching complexity in both GABAergic interneurons cultured from the medial ganglionic eminence (MGE) isolated from E14.5 mice as well as E18 primary hippocampal cultured neurons (36). While overexpression of liprin-α1 did not affect dendritic branching in either study (13, 36), overexpression partially rescued the deficit in dendritic branching following liprin-α1 knockdown in cultured hippocampal neurons (36). Even though GRIP plays a role in dendritic growth *via* regulating EphB surface expression (38), the expression of a liprin-α1 isoform unable to bind GRIP did not affect dendritic growth (20).

### Spinogenesis

Knocking down liprin-α1 decreased protrusions in both hippocampal culture neurons and *in vivo* mouse sections, indicating liprin-α1 promoted synapse formation (36), even though overexpression of liprin-α1 does not affect spine density (39). Together, these data suggest liprin-α1 is not sufficient for synapse formation. Liprin-α1 phosphorylation at threonine 701 (T701) by Cdk-5 negatively regulated liprin-α1 function in both spine and PSD-95 density, with phosphorylated liprin-α1 preferentially localized away from the postsynaptic density (36). Moreover, during neuronal development, increased spine formation in cultured neurons is correlated with increased liprin-α1 synaptic localization and decreased T701 phosphorylation (36). To further test the role of liprin-⍺1 T701 phosphorylation, researchers designed a phospho-interfering peptide composed of a T701-containing peptide (15 amino acids) from liprin-α1 fused to a cell-penetrating peptide (16 amino acids). Treating cultured neurons with the phospho-interfering peptide successfully decreased liprin-⍺1 T701 phosphorylation and subsequently increased synaptic localization of both liprin-⍺1 and PSD-95 within one hour. However, liprin-α1 T701 phosphorylation did not affect dendritic branching (36), suggesting liprin-α1 function in dendritic branching and spine formation is mediated by independent liprin-⍺1 interactors.

### AMPAR trafficking

**Figure 4:**
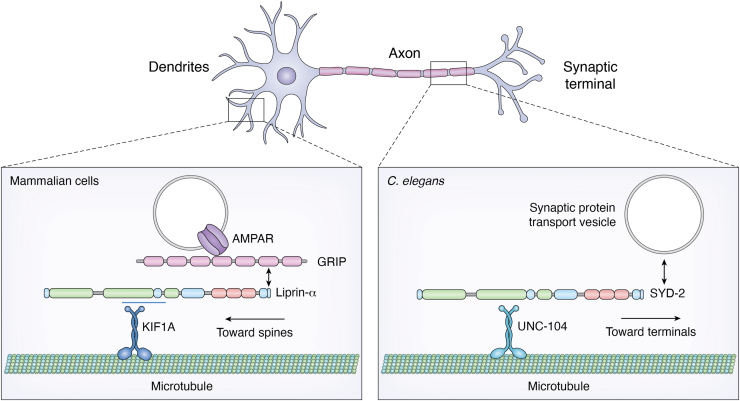
**Liprin-α role in trafficking.** Simplified schematic showing liprin-α/SYD-2 role in trafficking. In *C. elegans*, SYD-2 binds to UNC-104, a kinesin motor protein, to promote anterograde transport of synaptic proteins. In mammalian cells, liprin-α binds directly to KIF1A and GRIP, providing a link between AMPARs and motor proteins to promote the trafficking of receptors and other liprin-α interactors to spines. Figure created using BioRender.

AMPA-sub type ionotropic glutamate receptors are localized to postsynaptic spines and are most known for driving fast excitatory synaptic transmission (40). Surface trafficking of AMPARs is a critical step for regulating its function. Liprin-α1 promotes the surface trafficking of AMPARs through direct interactions with GRIP, a multi-PDZ domain protein that binds AMPARs, and KIF1A, a kinesin motor protein (19, 20) (Fig. 4). Additionally, liprin-α homologs contribute to axonal transport in invertebrate models through UNC-104, a KIF1A homolog (41). Researchers hypothesized liprin-α1 could link vesicles containing AMPARs to motor proteins to facilitate synaptic trafficking. Mechanistically, liprin-α also interacts with GIT1, a GTPase with roles in membrane trafficking and actin cytoskeleton regulation to regulate AMPAR trafficking (9). Inhibition of the direct interaction between GIT1 and liprin-α1 significantly decreased the surface clustering of AMPARs. Expression of the liprin-α1 splice isoform lacking the C-terminal PDZ binding motif necessary for GRIP binding decreased both AMPAR trafficking and spine formation, suggesting liprin-α proteins coordinate synapse formation and AMPAR trafficking to spines (20).

### Synaptic plasticity

Knocking down liprin-α1 significantly attenuated NMDA receptor (NMDAR)-dependent long-term potentiation (LTP) while introducing a liprin-α phospho-interfering peptide facilitated LTP (36). Presumably NMDAR signaling decreased liprin-α1 T701 phosphorylation. Interestingly, liprin-α1-GRIP interaction is important for muscarinic acetylcholine receptor mediated long-term depression (mAchR-LTD), but not mGluR-LTD, *via* regulating AMPAR endocytosis (37). Whether and how liprin-α1 regulates AMPAR exocytosis/endocytosis in response to LTP and NMDAR-LTD stimuli, respectively, are not yet reported.

## Postsynaptic signaling downstream of liprin-α1

As described above, GRIP, GIT1, and KIF1A mediated liprin-α1 function in AMPAR trafficking, and liprin-α1 acted in mAchR-LTD through GRIP, however, the mechanism involved in liprin-α1 promoting dendrite growth and spine formation is not clear.

### GIT1

Among the liprin-α1 interacting proteins, GIT1 is known to play critical roles in both dendritic growth (42) and synapse formation (43). Expression of a dominant negative GIT1 mutant (44), GIT1 knockdown (45) or GIT1 knockout (46, 47, 48, 49) decreased neurite outgrowth, spine density, synaptic plasticity, and/or led to a cognitive deficit. Moreover, proteins within the GIT interaction network and downstream F-actin remodeling signaling cascades, such as β-PIX, RAC3, and PAK, also contribute to neurite outgrowth and spine density (45). However, the only data linking liprin-α1 and GIT1 in dendrite growth that the authors identified is that expression of liprin-α1 mutant unable to bind to GIT1 decreased dendritic branching. Specifically, the number of intersections 20, 30, and 40 μm from cell body *via* a Sholl analysis were decreased (35). There is no data linking GIT1 to the role of liprin-α1 in spine formation. It is worth noting that liprin-α1 T701 phosphorylation only regulated spine formation, but not dendritic branching (36) suggesting the possibility of different mechanisms for liprin-α1 function in dendritic growth and spine formation.

### LAR family members

LAR and its family members, PTPσ and PTPδ, are strong candidate proteins that potentially mediate, or contribute to, liprin-α1 function in dendrite growth and spine density. Knocking down LAR family members (individually or in combination) and the expression of a LAR dominant negative mutant unable to bind to liprin-α1 led to a deficit in dendrite growth and spine formation (13, 20, 39). However, there was no significant effect of LAR family members on dendrite growth and spine formation in LAR family triple knockout neurons (50). This discrepancy could be due to the potential off-target effect of knockdown short hairpin RNA (51), dominant negative mutants interfering with other signaling pathways, and/or the unlikely compensatory pathway in triple knockout neurons.

### Liprin-β1

Liprin-β1 plays a role in photoreceptor axon path finding and neuromuscular junction synapse formation in *drosophila* (52) but there is no evidence for liprin-β1 functioning in conjunction with liprin-α1.

### Liprin-α1 synaptic localization

Liprin-α1 can localize to postsynaptic spines, however, the specific protein interaction that mediates liprin-⍺ synaptic localization has not been determined. Liprin-α1 forms a complex with PSD-95 (36), yet additional experiments suggest this interaction is indirect (Mayer and Xia, not published observation). Consistently, liprin-α family proteins contain a class-II PDZ ligand, whereas PSD-95 contains class-I PDZ domains. Liprin-α family proteins directly interact with GRIP *via* PDZ6 (20); however, GRIP does not concentrate in synaptic spines. One possible mechanism mediating liprin-α spine localization is through binding to GIT1 (Fig. 1*A*). GIT1 localizes to synapses through the direct interaction with β-PIX which binds to Shank3. Additionally, β-PIX binding to Shank3 was required for recruiting GIT1-β-PIX condensates to synapses (53). Future experiments are needed to determine whether inhibiting liprin-α1 binding to GIT1 prevents liprin-⍺1 localization at synaptic spines.

## Liprin-α1 regulates focal adhesion dynamics and cell motility

**Figure 5:**
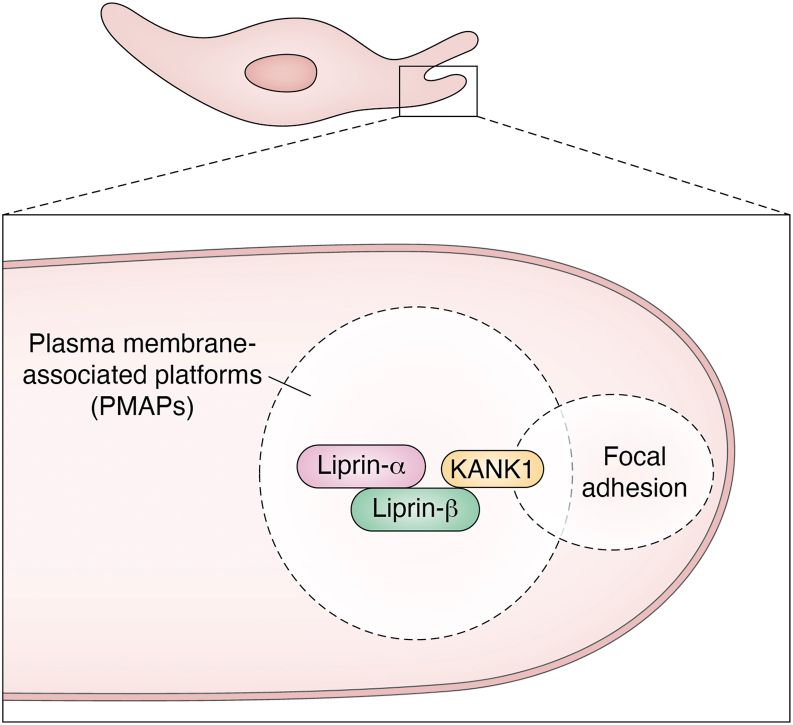
**Liprin-α1 role in cell adhesion.** Simplified schematic of liprin-α1 localized to plasma-membrane-associated platforms (PMAPs) adjacent to focal adhesions through the direct interaction with liprin-β and indirect interaction with KANK1. Figure created using BioRender.

Liprin-α1 is ubiquitously expressed and performs diverse functions beyond the nervous system, including roles in cell adhesion (24), motility (26, 27), and cell spreading (54, 55). Focal adhesions, which connect the actin cytoskeleton to the extracellular matrix, are critical for adhesion-dependent cell migration through their dynamic assembly and disassembly (1, 56). These structures are formed by integrin receptors, which cycle between active and inactive conformations, with active integrins exhibiting higher ligand affinity and requiring clustering for migration (57). Liprin-α1 localized to plasma membrane-associated platforms (PMAPs) adjacent to focal adhesions (58) (Fig. 5). The scaffold protein KANK1 facilitated PMAP localization around focal adhesions *via* interactions with key focal adhesion components, vinculin and paxillin (59). Liprin-β interacted with both KANK1 and liprin-α1, linking these scaffolds. Liprin-α1 contributed to the stabilization of integrin receptors at the cell surface in chicken embryo fibroblasts (55), and, in concert with ERC1/ELKS, it regulated focal adhesion turnover by promoting stabilization of active integrins.

Liprin-α1 has been implicated in cancer cell migration and invasion due to its role in cell motility. Located at the frequently amplified 11q13 locus in epithelial cancers, liprin-α1 is upregulated in breast cancer cells (1, 26). Its influence on cancer cell behavior appears context-dependent: in non-invasive cell lines, liprin-α1 promoted proliferation, whereas in invasive lines, it primarily facilitated invasion (26, 60). In breast cancer cells, liprin-α1 is essential for maximal migration and invasion, partly through stabilizing lamellipodial protrusions necessary for effective motility. Additionally, liprin-α1 modulates invadopodia formation and extracellular matrix degradation, further supporting its role in promoting invasive behavior. These studies highlight liprin-α1 function in oncogenic signaling pathways across diverse cancer models.

## Liprin-α1 regulates fibronectin fibrillogenesis and vascular morphogenesis

In primary endothelial cells, liprin-α1 is localized in proximity to fibrillar adhesions and knocking down liprin-α1 reduced active α5/β1 integrin recycling (16). The specific effect of liprin-α1 on the recycling of active α5/β1 integrin, but not total α5/β1 integrin, presumably resided in the ability of liprin-α1 to interact only with active α5/β1 integrin *via* the unclasped β1 integrin intracellular domain. The recycled active α5/β1 integrin brought endogenous fibronectin and its associated active integrin complex *via* post-Golgi carrier vesicles to fibrillar adhesion sites for exocytosis. The trans-Golgi network (TGN) exit of fibronectin and its subsequent polymerization are controlled by liprin-α1, LAR (or PTPRF), PI4K, Rab11 and AP-1a (16), however, the signaling causality among these proteins in promoting fibronectin TGN exit is not entirely clear. Moreover, LAR, PI4K and AP proteins are all important for active integrin recycling as well as fibronectin secretion and polymerization. It was suggested that liprin-α1 may function in the TGN exit of the active integrin-fibronectin complex; however, the relationship between liprin-α1, LAR and PI4K in this process is not clear. The seemingly different results regarding the role of liprin-α1 in the regulation of integrin stabilization *versus* recycling could be explained by differences in methodological approaches and cell types: Asperti *et al.* utilized a monoclonal antibody recognizing total β1-integrin to study receptor endocytosis in chicken embryo fibroblasts (55). Therefore, increased endocytosis following liprin-α1 downregulation and decreased endocytosis after liprin-α1 overexpression can be interpreted as liprin-α1 stabilizing total β1-integrin at the cell surface or increasing total β1-integrin recycling to cell surface. On the other hand, Mana *et al.*, (16) utilized an assay to directly quantify the recycling of active α5β1-integrin complexed with fibronectin in primary endothelial cells.

## Liprin-α liquid-liquid phase separation

Efficient spatiotemporal signaling relies on compartmentalization, often *via* membrane-bound domains, but some critical cellular regions like the presynaptic active zone, postsynaptic density, and focal adhesions, lack membranes and are enriched in specific proteins (61, 62). These membrane-less compartments, also called condensates, form dynamic, high-order protein assemblies essential for synaptic transmission and cell motility. They are thought to arise through liquid-liquid phase separation (LLPS), where proteins demix to form condensates that exchange with the cytosol. Within the condensate, proteins can perform active functions. Additionally, the condensed phase can also act as a mechanism to sequester a protein away from a subcellular compartment, inhibiting function. Protein oligomerization and coiled-coil domains facilitating weak interactions are commonly found in proteins that undergo LLPS (63). Core scaffolds such as liprin-α and its partners (ELKS/ERC1, RIM, GIT1, liprin-β1, LAR) undergo phase separation, driving the assembly of these specialized compartments (23, 31, 53, 64).

Liprin-α2 can form condensates *via* interactions with binding partners through the oligomerization of its N-terminal coiled-coil domain (65). This oligomerization promotes ELKS phase separation and spatially regulates ELKS distribution by preventing its incorporation into RIM/RIM-BP condensates which contain voltage-gated calcium channels (34). Interestingly, liprin-α2 overexpression alone forms insoluble aggregate, *i.e.*, droplet-like structures that show no fluorescence recovery after photobleaching (31, 66).

Unlike liprin-α2, which forms irreversible aggregates, liprin-α3 and the *C. elegans* homolog SYD-2 undergo dynamic, reversible phase separation regulated by phosphorylation (31, 67). In HEK293 cells, phosphorylation of liprin-α3 at S760 by PKC enhanced its phase separation (31). Co-expression with active zone proteins RIM and Munc13-1 promoted condensate formation at the plasma membrane, which further increases in size upon PKC activation. When liprin-α2 and liprin-α3 are co-expressed, they formed composite condensates, with liprin-α2 enriched in the core and liprin-α3 at the periphery—a spatial organization reminiscent of phase-separated structures formed by TARP γ8, PSD-95, and SynGAP (68). Fluorescence recovery after photobleaching (FRAP) confirmed liprin-α3 exhibited recovery, indicating dynamic exchange, whereas liprin-α2 remained static (31). PKC activation further enhanced the co-localization of both isoforms within shared condensates. Similarly, SYD-2 phase separation was promoted by phosphorylation through SAD-1 kinase (67). Phosphorylation at three sites near the first SAM domain relieved autoinhibition by disrupting intramolecular interactions between the N-terminus and the SAM domains, enabling SYD-2 condensation and subsequent active zone assembly (67).

Phosphorylation also promotes liprin-α1 phase separation (23). Specifically, preventing dephosphorylation *via* mutating the PPP2R5D binding site (short linear motif 4 (SLiM4)) on liprin-α1 promoted liprin-α1 phase separation. Phosphoproteomic data indicated that liprin-α1 phosphorylation at many sites was affected by the SLiM4 mutation. Liprin-α1 S763, located in liprin-α1 intrinsically disordered region, was confirmed to be a PPP2R5D holoenzyme site and phosphorylation of this site promoted liprin-α1 phase separation. Even though phosphorylation of liprin-α1 and SYD-2 both promoted their phase separation, the mechanisms diverge as deletion of SAM domains in liprin-α1 did not lead to constitutive phase separation as with SYD-2 (23, 67). Instead, liprin-α1 mutant unable to bind to liprin-β1 promoted its phase separation. Consistent with this, conditions promoting liprin-α1 phase separation showed a decreased interaction between liprin-α1 and liprin-β1. The study suggested liprin-α1 phosphorylation leads to decreased interaction with liprin-β1 which results in subsequent phase separation. Whether liprin-α3 phosphorylation utilizes the same mechanism in phase separation is not known, nor are the identity of the kinase phosphorylating liprin-α1 S763 and the phosphatase for liprin-α3 S760.

## Liprin-α1 phospho-regulation by kinases and phosphatases

**Figure 6:**
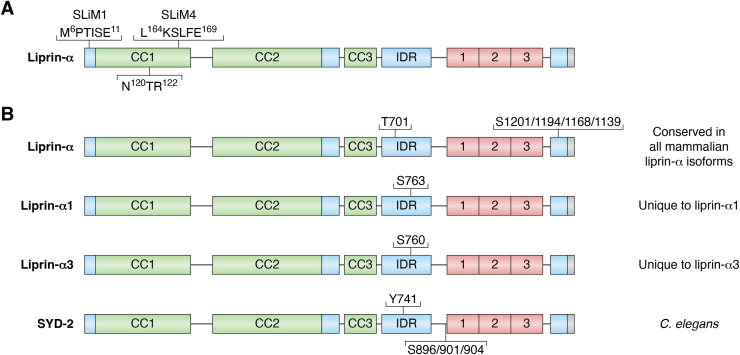
**Phosphatase binding motifs and phosphorylation sites on liprin-α proteins.***A*, PPP2R5C binds SLiM1 on liprin-α, while PPP2R5D binds to SLiM4. Residue numbers a sequence for each SLiM are noted. Residues N^120^TR^122^ were also identified as required for both PPP2R5C and PPP2R5D binding. *B*, phosphorylation sites on liprin-α proteins. Threonine 701 (T701) is phosphorylated by Cdk5, and Serine 1201, 1194, 1168, and 1139 (S1201/1194/1168/1139) are consensus sites for CaMKII. Figure created using BioRender.

Phosphorylation regulates liprin-α1 in both neuronal and non-neuronal cells (Fig. 6*B*). Neuronal activity tightly regulates synapses, and liprin-α1 is regulated *via* activity-dependent calcium signaling and Cdk-5-mediated phosphorylation (13, 36). While decreased neuronal activity *via* tetrodotoxin treatment increased liprin-α1 protein expression and clustering, increased neuronal activity *via* picrotoxin treatment had the opposite effect (13). This phenomenon was regulated *via* CaMKII activity which is likely positively regulated by neuronal activity. Regulation of liprin-α1 required its C-terminus containing four CaMKII sites (Fig. 6*B*) and the PEST motif, a sequence targeting proteins to proteolytic degradation. Expression of liprin-α resistant to degradation impaired dendritic morphogenesis likely caused by the disruption of LAR surface trafficking. Overall, regulation of liprin-α levels *via* CaMKII-mediated degradation promoted proper LAR surface targeting and dendrite development. However, the authors did not generate site-specific phospho-antibodies to definitively demonstrate that the putative sites on liprin-α1 are indeed phosphorylated by CaMKII. In addition to regulation *via* CaMKII, liprin-α1 is phosphorylated by Cdk5 in response to neuronal activity (36). Phosphorylation of liprin-α1 at T701 was decreased following increased neuronal activity, likely due to Cdk5 activator p35 degradation (69). Phospho-null mutation of T701 increased spines and PSD-95 puncta, implicating liprin-α1 in synapse development. Neuronal activity inhibited Cdk5-mediated phosphorylation of liprin-α1 and, in return increased the association between liprin-α1 and PSD-95, enhancing the synaptic localization of PSD-95. Additionally, in non-neuronal cells, DYRK3 phosphorylates liprin-α1 to negatively regulate PMAPs, but the exact site(s) on liprin-α1 phosphorylated by DYRK3 are not known (70).

Liprin-α1 T701 is conserved in all mammalian liprin-α isoforms, however, whether the other isoforms are phosphorylated at T701 and the function of T701 phospho-regulation has not been studied. On the other hand, liprin-α3 is phosphorylated by PKC at serine 760 (S760), a site not present in other liprin-α isoforms (31) (Fig. 6*B*). Phosphorylation of liprin-α3 at S760 increased its interaction with RIM and increased localization at the active zone to promote neurotransmitter release (31). Interestingly, liprin-α1 was shown to be phosphorylated at S763, a site unique to liprin-α1 itself, and phosphorylation of this site on liprin-α1 decreased its interaction with liprin-β1 (23) (Fig. 6*B*). *C. elegans* liprin-α homolog SYD-2 is phosphorylated at three sites in or around the first SAM domain, and these phosphorylation events are critical for the role SYD-2 plays in synapse formation *via* preventing the three SAM domains from interacting with N-terminal fragment (67) (Fig. 6*B*). Similar to the three serine/threonine phosphorylation sites on SYD-2 leading to SYD-2 open conformation, LAR-RPTP (called PTP3 in *C. elegans*) dephosphorylated SYD-2 at Y741, keeping SYD-2 in a closed conformation (71). In the PTP3 mutant, SYD-2 phosphorylation at Y743 is increased, leading to increased interaction between SYD-2 and kinesin motor KIF1A (called UNC-104 in *C. elegans*). Even though these sites are conserved in mammalian liprin-α proteins, whether they are phosphorylated and the role of these phosphorylation sites in mammalian cells is not known.

Among all liprin-α phosphorylation sites, except S763 of liprin-α1 and Y741 on SYD-2, the responsible phosphatase is not known even though all liprin-α isoforms contain conserved SLiM1/4 motifs and can bind to LAR and its family members PTPσ and PTPδ.

## Liprin-α1 mediates the dephosphorylation of its interaction partners *via* PPP2R5C/D binding

Liprin-α1 forms a complex with protein phosphatase 2A (PP2A) (17, 21) and this complex formation is mediated by two PP2A regulatory subunits, PPP2R5C (18, 21, 72) and PPP2R5D (18, 23, 73), and there is evidence that this interaction is multifaceted. The PPP2R5C/D holoenzymes can directly mediate liprin-α1 function through regulating liprin-α1 phosphorylation (23), and the PPP2R5C/D holoenzymes can dephosphorylate other liprin-α1 binding proteins (18, 22). PPP2R5C/D are members of the B′ family of regulatory subunits responsible for mediating PP2A substrate specificity (74). The B′ family recognizes a SLiM on its substrates, termed the LxxIxE motif (75, 76). This motif recognition is highly conserved among B′ family binding proteins and is found in intrinsically disordered regions. Liprin-α1 has eight potential LxxIxE motifs which we termed SLiM1-8 (23), and it was determined that liprin-α1 SLiM1 mediates its interaction with PPP2R5C while SLiM4 mediates the interaction with PPP2R5D (23) (Fig. 6*A*). Liprin-α1 localized to cell structures adjacent to focal adhesions called PMAPs, and this localization was not affected by PPP2R5D, however, liprin-α1 function in cell spreading, invasion, motility, and lamellipodia dynamics were mediated by PPP2R5C (72). Loss of liprin-α1 or PPP2R5C function decreased cell migration and invasion. Liprin-α1 overexpression attenuated the loss of PPP2R5C. Lastly, liprin-α1 WT, but not liprin-α1 mutant unable to bind to PPP2R5C, rescued the deficit in cell spreading from the loss of liprin-α1. In another report, researchers provided evidence that PPP2R5C could counteract the dual specificity kinase 3 (DYRK3) in regulating liprin-α1 function *via* regulating its phosphorylation status (70), however, the exact site(s) on liprin-α1 for the modulation is not yet known.

Liprin-α1 binding to PP2A was necessary for liprin-α1-mediated promotion of sonic hedgehog signaling in primary cilia, a microtubule-based protrusion from cell body (17). Sonic hedgehog binds to patched receptors, leading to movement away from cilia, allowing smoothened to traffic into cilia. Smoothened signaling leads to KIF7 translocation to the tip of the cilia, helping to orchestrate the proteolytic processing of transcription factor Gli2/3 to the active form before its movement to the nucleus for gene transcription. Liprin-α1 binds PP2A, facilitating the dephosphorylation of KIF7 at S1337, which is required for the KIF7 localization to the tip of the cilia (17). Interestingly, liprin-α1 localizes at the base of the primary cilia and its binding to KIF7 is enhanced by hedgehog signaling (17). While dephosphorylated KIF7 translocated to the cilia tip, liprin-α1 localization was unchanged. How hedgehog signaling promotes liprin-α1-KIF7 interaction and whether KIF7 S1337A has decreased binding to liprin-α1 are not known.

Liprin-α1 binds to CEP170B, a microtubule minus-end-binding protein which negatively regulates microtubule minus-end distribution and is required for directional vesicle trafficking (18). The authors found that liprin-α1 complexed with both PPP2R5C and PPP2R5D, and moreover, they found two point-mutations (N^120^TR^122^ → ATA) on liprin-α1 that abolished its interaction with PPP2R5C/D separate from the LxxIxE interaction interface (75) (Fig. 6*A*). The liprin-α1 (A^120^TA^122^) mutant did not affect liprin-α1 interaction with CEP170B, allowing them to examine the effect of PPP2R5C/D holoenzyme complex on CEP170B (18). They first demonstrated that liprin-α1 was required for CEP170B localization to microtubules *via* liprin-α1 knockout, even though liprin-α1 itself was not localized to microtubules. Next, they used a molecular replacement strategy (transfection of liprin-α1 WT or ATA mutant into the liprin-α1 knockout background) to demonstrate that liprin-α1 WT, but not its PPP2R5C/D binding deficient mutant, rescued the CEP170B microtubule localization. These data suggest dephosphorylation of CEP170B proceeds microtubule localization. This mechanism, where liprin-α1 “catches” the PP2A substrate CEP170B, then facilitates dephosphorylation before releasing the interactor, is reminiscent of KIF7 signaling in cilia (17).

In these cases, liprin-α1, as a scaffolding protein, binds to both the PPP2R5C/D holoenzyme and CEP170B/KIF7, thus bringing the phosphatase and substrate into close physical proximity, increasing substrate specificity and catalytic efficiency. As discussed above, liprin-α1 binds to many other proteins playing roles in different cellular processes such as synapse formation, focal adhesion, and cell motility. Whether liprin-α1 brings PP2A near other interactors such as RIM, GIT1, LL5β, ELKS/ERC1, GRIP, and activated integrin β1 to mediate dephosphorylation is not known. However, there is ample literature suggesting liprin-α1 function is modulated by its own phosphorylation status. In this case, liprin-α1 acts as both a PPP2R5C/D-PP2A binding protein and a substrate.

## Potential methodological issues with the published literature related to liprin-α proteins

Protein localization and function are intimately linked; However, proteins, including the liprin-α family, can directly or indirectly effect interactors to exert functions in separate subcellular compartments. For example, liprin-α1 can regulate microtubule dynamics and vectorial transport even though it does not localize to microtubules (18). Additionally, liprin-α1 function is necessary for KIF7 localization to the cilia tip despite liprin-α1 localizing away from the tip (17). Therefore, the function of a protein in a cellular process cannot be used to conclude that it is localized to the corresponding subcellular compartment. Interestingly, recombinant liprin-α1 and LAR, when expressed individually, localized to both the dorsal and ventral sides of COS7 (77); however, co-expression of liprin-α1 and LAR led to increased ventral localization of both proteins, suggesting a synergistic interaction between liprin-α1, LAR, and adherent junction proteins. This may also extend to liprin-α1 function in localizing its interaction partner, unclasped or active α5β1 integrin, preferentially to the basolateral side in primary endothelial cells (16). Protein localization and function need to be studied separately to determine whether the effect is direct, and/or whether the function of the same protein has independent functions in different cellular compartments.

Liprin-α proteins play important roles in a wide range of cellular processes and the localization of liprin-α in different cell types has been studied extensively by many different laboratories. However, protein localization studies rely on the availability of specific antibodies and only the knockout control can guarantee the authentic localization of proteins studied. Earlier studies utilizing liprin-α antibodies to study protein localization without stringent knockout controls should not be used to conclude information about specific isoform localization. For full confidence of the relative liprin-α isoform localization in various cellular compartments, such as presynaptic terminals, dendrites, and even spines, needs to be determined using validated specific antibodies with knockout controls, commercially known tag antibodies combined with CRISPR knock-in of tags into the locus of PPFIA1-4, the genes encoding liprin-α, and/or complemented from other studies, such as biochemical fractionations.

Overexpression studies provide strong evidence for the function of a protein, but subsequent loss-of-function experiments are required to confirm conclusions and account for protein overexpression artifacts. Moreover, interpretating results from overexpressing mutant proteins even with congruent loss-of-function studies requires extra care as mutant proteins could have unintended effects on protein interactions or signaling cascades. Cell phenotypes resulting from knocking down endogenous proteins *via* the expression of short hairpin RNA (shRNA) require rescue experiments where there is simultaneous expression of recombinant wild type protein (with silent mutations to resist shRNA effect) as shRNA can have off-target effects (51).

## Conclusion and prospects

Liprin-α family proteins are important for many cellular processes, likely mediated by their interacting proteins. The liprin-α family are phosphoproteins, modulated by various kinases and phosphatases to ultimately regulate liprin-⍺ function. Liprin-α1 is ubiquitously expressed and still requires more research to determine its function in a wider range of cellular contexts. The distinct *versus* redundant roles of liprin-α family proteins in the same cellular processes also need to be further defined. A prime example is liprin-⍺ function in the presynaptic terminal. Liprin-α1-4 are expressed presynaptically, and they perform redundant functions in regulating baseline neurotransmitters, such as glutamate release. However, individual liprin-⍺ isoforms have distinct phosphorylation sites, suggesting different modulators may affect glutamate release *via* different liprin-⍺ isoforms. In this regard, the liprin-α1-4 quadruple knockout cell line (29) is a useful tool for investigating the similarities and differences between liprin-⍺ isoform synaptic function.

## Conflict of interest

The authors declare that they have no conflicts of interest with the contents of this article.
